# A new family of uncultivated bacteria involved in methanogenesis from the ubiquitous osmolyte glycine betaine in coastal saltmarsh sediments

**DOI:** 10.1186/s40168-019-0732-4

**Published:** 2019-08-27

**Authors:** Helen J. Jones, Eileen Kröber, Jason Stephenson, Michaela A. Mausz, Eleanor Jameson, Andrew Millard, Kevin J. Purdy, Yin Chen

**Affiliations:** 1 School of Life Sciences, University of Warwick, Coventry, CV4 7AL UK; 2 Microbial Biogeochemistry, RA Landscape Functioning, ZALF Leibniz Centre for Agricultural Landscape Research, 15374 Müncheberg, Germany; 3 Department of Infection, Immunity and Inflammation, University of Leicester, Leicester, LE1 7RH UK

## Abstract

**Background:**

Coastal environments are dynamic and rapidly changing. Living organisms in coastal environments are known to synthesise large quantities of organic osmolytes, which they use to cope with osmotic stresses. The organic osmolyte glycine betaine (GBT) is ubiquitously found in marine biota from prokaryotic Bacteria and Archaea to coastal plants, marine protozoa, and mammals. In intertidal coastal sediment, GBT represents an important precursor of natural methane emissions and as much as 90% of total methane production in these ecosystems can be originated from methanogenesis from GBT and its intermediate trimethylamine through microbial metabolism.

**Results:**

We set out to uncover the microorganisms responsible for methanogenesis from GBT using stable isotope labelling and metagenomics. This led to the recovery of a near-complete genome (2.3 Mbp) of a novel clostridial bacterium involved in anaerobic GBT degradation. Phylogenetic analyses of 16S rRNA gene, functional marker genes, and comparative genomics analyses all support the establishment of a novel family *Candidatus* ‘Betainaceae’ fam. nov. in *Clostridiales* and its role in GBT metabolism.

**Conclusions:**

Our comparative genomes and metagenomics analyses suggest that this bacterium is widely distributed in coastal salt marshes, marine sediments, and deep subsurface sediments, suggesting a key role of anaerobic GBT metabolism by this clostridial bacterium in these ecosystems.

**Electronic supplementary material:**

The online version of this article (10.1186/s40168-019-0732-4) contains supplementary material, which is available to authorized users.

## Background

Coastal marine environments represent one of the largest dynamic and productive ecosystems on Earth which supports a third of the world’s populations [1]. This environment experiences daily fluctuations in a range of environmental conditions, including water levels, salinity, and temperature. Organisms living in the dynamic coastal environment cope with changing environmental conditions by synthesising a range of organic and inorganic osmoprotectants (osmolytes) in order to cope with water stress [2–4]. A ubiquitous organic osmolyte, which is produced by both prokaryotic and eukaryotic marine organisms, is glycine betaine (GBT). Many coastal marine organisms can accumulate GBT, and as high as 1 M intracellular GBT concentrations have been reported in some microbes living in hypersaline environment [5]. Once released, GBT catabolism contributes to methane formation through anaerobic microbial metabolism. Globally, coastal marine environment accounts for three fourths of oceanic methane emissions and recent assessment suggests ~ 13 Tg methane year^−1^ from coastal environment [6]. Previous estimations in microcosms using coastal marine sediments suggest that up to 90% of methane emissions can be resulted from the degradation of GBT and other structurally related quaternary amine compounds [7, 8].

Although GBT plays an important role in methane cycle in coastal sediments, the identity of the microorganisms responsible for GBT-dependent methanogenesis is still poorly understood [8–10]. In the intertidal sediment in Maine, USA, GBT was converted by sulfate reducers to trimethylamine (TMA) followed by methanogenesis although the identity of the microbes involved in GBT degradation was not studied [8]. It was later shown by Heijthuijsen and Hansen [11] that the sulfur-reducing bacterium *Desulfuromonas acetoxidans* can degrade GBT to produce TMA and acetate, some of which was further oxidised to produce reductant for the initial reduction of GBT. Whether or not sulfate reducers are indeed involved for GBT degradation in coastal sediments remains elusive. Heijthuijsen and Hansen [12] subsequently isolated sulfate reducers of the *Desulfobacterium* genus which converted GBT to dimethyglycine instead of TMA. More recently, methanogens in the *Methanococcoides* and *Methanolobus* genera have been shown to produce methane through direct demethylation of GBT, yielding dimethylglycine as the by-product [9, 13].

In this study, we set out to characterise the microorganisms involved in methanogenesis from GBT in coastal salt marsh sediments using a synthesis of DNA-stable isotope labelling coupled with metagenomics sequencing and assembly to retrieve near-complete metagenome-assembled genomes (MAGs) of the microorganisms responsible for the degradation of ^13^C-isotope-labelled GBT. A unique advantage of MAGs derived from stable isotope-labelled ^13^C-DNA is to allow linking microbial identity to metabolic function. Using this approach, we show in this study the recovery of a near-complete genome (2.3 Mbp) of a non-sulfate-reducing clostridial bacterium involved in anaerobic GBT degradation in a coastal salt marsh sediment. Phylogenetic analyses, metabolic reconstruction from MAGs, and comparative genomics analyses support the establishment of a novel family *Candidatus* ‘Betainaceae’ fam. nov. involved in methanogenesis from GBT. These bacteria appear to be widely distributed in coastal sediments, salt marshes, and deep subsurfaces as demonstrated by genome mapping using metagenomics recruitment.

## Results

### Methanogenesis from GBT in salt marsh sediments and microbial community sequencing of 16S rRNA genes

**Fig. 1:**
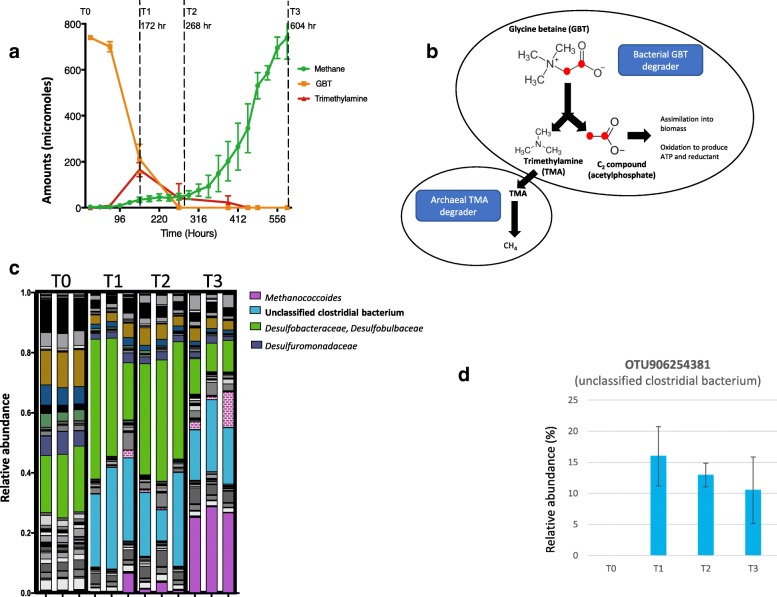
A novel clostridial bacterium involved in glycine betaine (GBT)-dependent methanogenesis from a salt marsh sediment. **a** Microcosm incubations of salt marsh sediments with the addition of GBT. Methane formation and GBT and trimethylamine (TMA) concentrations in the sediment slurry were measured. Microcosms were set up in three biological replicates, and error bars represent standard deviations. Samples were taken from the microcosms at T0, T1, T2, and T3 for amplicon sequencing analyses. **b** A working model of GBT degradation by bacterial degraders which produce TMA and a two-carbon compound, and archeal methanogens which produce methane from TMA. The red dots highlight the carbon atoms in GBT which are labelled with ^13^C. **c** Miseq amplicon sequencing of microbial 16S rRNA genes during GBT-dependent methanogenesis. **d** Increase of relative abundance of this novel clostridial bacterium over time in the GBT-amended microcosms

We sampled the Stiffkey salt marsh in Norfolk, UK, and set up microcosm incubations using the most active layer (1.5–4.5 cm off the surface) of the salt marsh sediment for methanogenesis. When left untreated, no methane formation occurred in 96 h. However, active methanogenesis occurred when the microcosms were amended with either GBT (Fig. 1a) or TMA (a potential intermediate in anaerobic GBT degradation pathway, Additional file 6: Figure S1). In the microcosms amended with GBT, the substrate rapidly disappeared and a spike of TMA is found before significant methane production started, suggesting that TMA was likely the intermediate of methanogenesis from GBT in this salt marsh sediment (Fig. 1b).

We sampled these GBT-amended microcosms at three time points (172 h, 268 h, 604 h), and the microbial community change over time was determined by amplicon sequencing of bacterial and archaeal 16S rRNA genes. Before enrichment with GBT (T0), the sediment had a diverse group of microbes, including *Gammaproteobacteria*, *Deltaproteobacteria*, *Epsilonbacteraeota*, *Bacteroidetes*, *Acidobacteria* and *Firmicutes* (Fig. 1c, Additional file 1: Table S1). After GBT amended to the microcosms, significant increase in relative abundance was seen in several OTUs that are assigned to three microbial clades—deltaproteobacterial *Desulfobacteraceae/Desulfobulbaceae*, archaeal *Methanococcoides*, and a group of unclassified clostridial bacteria represented by OTU906254381 (Fig. 1d).

**Fig. 2:**
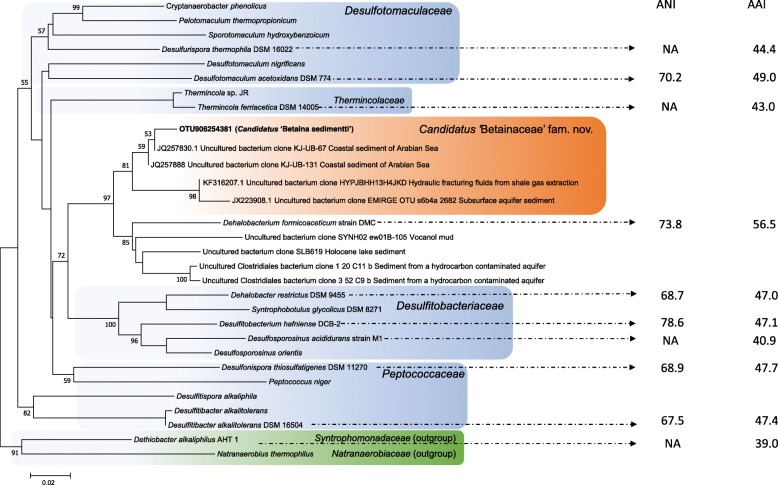
Comparative genomics analysis showing the average nucleotide identity (ANI) and average amino acid identity (AAI) values of this novel clostridial bacterium (bin 4, see Table 1) compared to the genome-sequenced bacteria in the order of *Clostridiales* [14]. The tree was constructed using 16S rRNA genes and phylogenetic analysis was conducted in MEGA7 [15]. The percentage of replicate trees in which the associated taxa clustered together in the bootstrap test (500 replicates) are shown next to the branches. The scale bar represents the number of substitutions per nucleotide. ANI and AAI values were calculated using the corresponding calculators as described in Rodriguez and Konstantinidis [16]. NA indicates that the ANI values are too close to the detection limit, and thus, no reliable values were returned

This unclassified group of clostridial bacteria (OTU906254381, MK313791) was barely detectable at T0 by amplicon sequencing of 16S rRNA genes, but their relative abundance increased significantly in the GBT-amended microcosms, accounting for up to 16% of all amplicon reads (Fig. 1d). The OTU sequence had 94% identity to the 16S rRNA gene of *Dehalobacterium formicoaceticum* and < 92% identity to that of any other cultivated bacteria in the JGI IMG ‘16S rRNA Public Isolates’ database, NCBI RefSeq Representative Genome Database, or the SILVA rRNA database. The most closely related environmental sequences were from uncultured bacteria (> 98% identity) retrieved from a variety of environments that are typically dynamic and can experience high salinity, including coastal marine sediments (JQ257830; JQ257888), subsurface aquifer sediments (KF316207), and shale gas extraction fracturing fluids (JX223908) (Fig. 2).

Amplicon sequencing analyses of the 16S rRNA genes also revealed that several OTUs belonging to the deltaproteobacterial *Desulfobacteraceae* were enriched by GBT addition, the most abundant of which (OTU822440212) had > 96% identity to the 16S rRNA gene of the *Desulfobacterium* (Additional file 6: Figure S2). The third group of abundant OTUs that was enriched was assigned to the methanogen *Methanococcoides*, which are known to utilise TMA as a substrate for methanogenesis [9, 17, 18]. Indeed, *Methanococcoides* became heavily enriched when the salt marsh sediments were incubated with TMA, accounting for more than 50% of the total microbial reads by time point 3 (Additional file 6: Figure S4).

### Recovery of population genomes of the novel clostridial bacteria from metagenome sequencing of ^13^C-stable isotope-labelled DNA

The data suggest that this unclassified clostridial bacteria represented by OTU906254381 are likely involved in the initial degradation of GBT to TMA, which is then further metabolised by the methylotrophic methanogen of the *Methanococcoides* to form methane in this salt marsh sediment (Fig. 1b), supporting a syntrophic interaction between Bacteria and Archaea in GBT-dependent methanogenesis [19].

To further support the role of this unclassified clostridial bacterium in GBT metabolism, DNA-stable isotope probing (SIP) microcosms were set up using 1,2-^13^C_2_-GBT and Miseq sequencing of 16S rRNA gene amplicons was performed on ‘heavy’ and ‘light’ fractions at three time points (T1, T2, T3). The data presented in Additional file 6: Figure S6a confirmed enrichment of this novel group of bacteria primarily in the heavy fractions. Similarly, DNA-SIP incubations using ^13^C_3_-TMA also confirmed the assimilation of ^13^C by *Methanococcoides*, hence confirming their role in methanogenesis from TMA (Additional file 6: Figure S6b). Therefore, the data from DNA stable isotope labelling and amplicon sequencing strongly suggest the carbon flow from GBT to TMA and subsequently TMA to methane by this novel group of clostridial bacteria and *Methanococcoides*, respectively (Fig. 1b).

In order to gain insight into the metabolism of this novel clostridial bacterium (OTU906254381), we chose the three biological replicates of heavy fractions of T2 from the ^13^C_2_-GBT SIP experiments for metagenome sequencing using the Illumina Hiseq platform. Metagenome reads were assembled and assigned into individual bins. This resulted in the assignment of a total of 148 bins, comprising of 20–28 bins from each ‘heavy’ fractions (microcosm replicates 1, 2, and 3) and 23–28 bins from each light fractions (Additional file 2: Table S2). Taxonomy assignment for each bin was performed by running against the RAST database, and MAGs with the highest quality (> 70% completeness and < 10% contamination) are shown in Table 1.

**Table 1:** Selected MAGs assembled from ^13^C_2_ glycine betaine stable isotope labelling microcosms

Sampleǂ	Bin	Taxonomy assignment by RAST		Length (bp)	Completeness (%)	Contamination (%)	*grdH*	*mttB*
		Class	Species					
Heavy_R1	4	*Clostridia*	*Thermincola* sp. JR	2.69E+06	94.5	2.3	+	
Light_R1	23	*Clostridia*	*Thermincola* sp. JR	2.88E+06	98.7	0.8	+	
Heavy_R1	5	*Clostridia*	*Clostridium thermocellum*	2.80E+06	73.5	4.7		
Light_R3	10	*Clostridia*	*Desulfotomaculum reducens*	5.28E+06	98.7	8.1	+	
Heavy_R3	21	*Clostridia*	*Thermincola* sp. JR	1.77E+06	78.5	3.0		
Heavy_R3	8	*Clostridia*	*Desulfotomaculum acetoxidans*	2.99E+06	97.8	2.5		
Light_R3	22	*Clostridia*	*Thermincola* sp. JR	2.13E+06	84.7	2.3		
Light_R2	5	*Clostridia*	*Thermincola* sp. JR	2.38E+06	76.0	1.3	+	
Heavy_R2	3	*Clostridia*	*Moorella thermoacetica*	2.81E+06	87.5	4.9		
Heavy_R2	21	*Deltaproteobacteria*	*Desulfobacterium autotrophicum*	6.49E+06	94.1	7.0		
Heavy_R3	22	*Deltaproteobacteria*	*Desulfobacterium autotrophicum*	6.62E+06	98.5	4.8		
Heavy_R3	5	*Deltaproteobacteria*	*Desulfobacterium autotrophicum*	5.81E+06	93.4	9.8		
Light_R3	26	*Deltaproteobacteria*	*Desulfuromonas acetoxidans*	2.61E+06	83.0	9.4		
Light_R1	27	*Deltaproteobacteria*	*Desulfuromonas acetoxidans*	2.11E+06	75.4	8.4		
Light_R1	10	*Deltaproteobacteria*	*Desulfuromonas acetoxidans*	3.62E+06	96.6	6.8		
Light_R3	12	*Deltaproteobacteria*	*Desulfuromonas acetoxidans*	2.72E+06	93.0	2.2		
Heavy_R3	28	*Deltaproteobacteria*	*Desulfuromonas acetoxidans*	2.41E+06	83.6	2.2		
Light_R2	9	*Deltaproteobacteria*	*Sorangium cellulosum*	1.48E+06	73.0	3.4		
Light_R3	14	*Gammaproteobacteria*	*Cellvibrio japonicus*	1.66E+06	72.9	2.4		
Heavy_R2	6	*Thermomicrobia*	*Thermobaculum terrenum*	1.81E+06	79.9	3.3		
Heavy_R1	6	*Euryarchaeota*	*Methanosarcina barkeri*	2.35E+06	75.8	1.0		+
Heavy_R3	2	*Euryarchaeota*	*Methanosarcina barkeri*	2.21E+06	74.3	0.0		+
Light_R1	18	*Euryarchaeota*	*Methanosarcina barkeri*	2.16E+06	74.6	1.3		+
Heavy_R2	7	*Euryarchaeota*	*Methanococcoides burtonii*	1.56E+06	74.0	1.3		+

Only MAGs with > 70% completeness and < 10% contamination are shown. *gdH* encodes for a glycine betaine reductase, and *mttB* encodes for a pyrrolysine-containing trimethylamine methyltransferase

^ǂ^‘heavy’ and ‘light’ DNA fractions from three independent biological replicates (R1, R2, R3) were sequenced and assembled

We focused our analyses on the MAGs that are assigned to *Clostridiales* by RAST because the 16S rRNA gene from this bacterium classified within this order (Fig. 1d) although near-complete genomes of MAGs related to *Desulfobacterium* and *Methanococcoides* were also retrieved (Table 1, Additional file 6: Figures S3 and S5). Out of the 9 bins that were assigned to *Clostridiales***,** 2 bins (Bin 4 and Bin 23) are nearly complete (94.5%, 98.7%) and had minimum estimation of contamination (< 5%) (Table 1). The genome sizes were 2.7 and 2.9 Mbp, obtained from 139 and 96 contigs, respectively. We performed comparative genome analyses of average nucleotide identity (ANI) and average amino acid identity (AAI) against closely related genomes in the order *Clostridiales*, and the data placed these two genomes in a novel clade (Fig. 2). The two genomes showed 56.5% AAI to the closely related bacterium *Dehalobacterium formicoaceticum* and between 40–50% AAI to other genomes of the order *Clostridiales*. We also performed phylogenetic analyses of the RpoB protein. The RpoB proteins from the two MAG bins are identical and showed 86% sequence identity to that of *Dehalobacterium formicoaceticum* and < 83% sequence identity to other genomes of the order *Clostridiales* (Additional file 6: Figure S7). Therefore, analyses of 16S rRNA gene, *rpoB* gene, and ANI analysis [20] all strongly suggest that this unclassified group of bacteria enriched by GBT forms a novel family within the *Clostridiales* order. We therefore propose the name *Candidatus* ‘Betaina sedimentti’ gen. nov., sp. nov. as the first representative of a new family, *Candidatus* ‘Betainaceae’ fam. nov. to encompass this novel uncultivated clostridial bacterium, suggesting its role in anaerobic GBT metabolism in salt marsh sediments.

### Metabolic reconstruction of *Candidatus* ‘Betaina sedimentti’ sp. nov. and its wide distribution in the environment

**Fig. 3:**
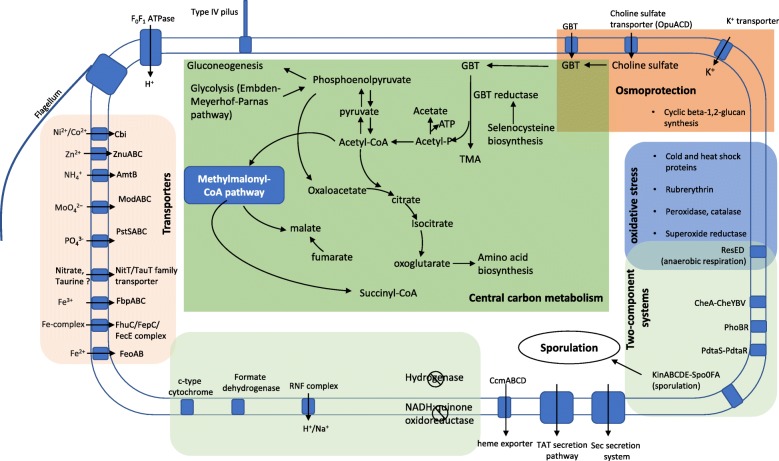
An overview of metabolic reconstruction of the key metabolism in *Canditatus* ‘Betaina sedimentti’. GBT, glycine betaine; TMA, trimethylamine

**Fig. 4:**
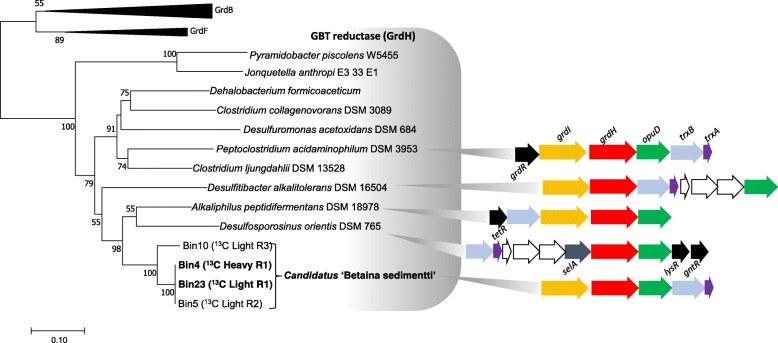
Phylogenetic analysis of the functional gene marker glycine betaine (GBT) reductase (GrdH) of *Canditatus* ‘Betaina sedimentti’, compared to glycine reductase (GrdB) and sarcosine reductase (GrdF). Boostrap values greater than 50% are shown. The scale bar represents substitutions per amino acid. *grdHI* encodes for the selenoprotein betaine reductase; *trxB* and *trxA* encode for thioredoxin reductase and thioredoxin, respectively; *opuD* encodes for a BCCT-type GBT transporter; *selA* encodes for selenocysteine synthase; *tetR*, *lysR* and *gntR* encode for transcriptional regulators

The near-complete genome sequences retrieved from the ^13^C_2_-GBT DNA-SIP-derived MAG provide an opportunity to explore the metabolic potential in this novel bacterium (Fig. 3). We found a complete gene set required for GBT reduction through the selenocysteine-containing betaine reductase (GrdHI), together with a BCCT-type GBT transporter (OpuD) and thioredoxin (TrxA) and thioredoxin reductase (TrxB) that are required for GBT uptake from the environment and electron transfer from NAD(P)H to GBT reductase, respectively (Fig. 4). GBT cleavage through betaine reductase produces acetyl-phosphate which is channelled to the central carbon metabolism through acetyl-CoA (Fig. 3), and complete gene sets for gluconeogenesis and glycolysis using the Embden-Meyerhof-Parnas pathway are present in the genome. Acetyl-phosphate is further converted to generate ATP, and the gene encoding an acetate kinase is found in its genome (Fig. 3, Additional file 5: Table S5). The TCA cycle is incomplete, and both the oxoglutarate dehydrogenase and the succinate dehydrogenase are missing from the genome. Instead, this bacterium appears to couple GBT reduction with amino acid fermentation through Stickland reaction which provides three-carbon intermediate for acetyl-CoA oxidation using the Methylmalonyl-CoA pathway [21]. The Methylmalonyl-CoA pathway provides essential intermediate such as malate and succinyl-CoA for anabolism.

Genome analysis suggests that *Candidatus* ‘Betaina sedimentti’ is unable to use sulfate as a terminal electron acceptor and the dissimilatory sulfate reduction pathway is absent. Lack of dissimilatory sulfate reduction pathway therefore makes this bacterium distinctly different from members of the *Desulfotomaculaceae*, *Desulfitobacteriaceae*, and *Peptococcaceae* (Fig. 2). Furthermore, the *Candidatus* ‘Betaina sedimentti’ genome does not encode the Wood-Ljungdahl pathway and is therefore distinct from members of the *Thermincolaceae* and the bacterium *Dehalobacterium formicoaceticum* (Fig. 2). *Thermincolaceae* and *Dehalobacterium formicoaceticum* are able to use the functional Wood-Ljungdahl pathway for autotrophic growth on one-carbon compounds, e.g. carbon monoxide and dichloromethane, respectively [22, 23].

Genome analysis also provides insights into the adaptation of this bacterium to the salt marsh environment. It contains several mechanisms of osmoprotection [2, 3] such as using potassium ions and membrane-derived oligosaccharides (e.g. cyclic glucans), as well as uptake and synthesis of compatible organic solutes (e.g. GBT biosynthesis from choline and choline sulfate). This bacterium also appears to have multiple mechanisms coping with oxidative stresses, and a complete sporulation pathway is also present. This versatility in adaptation to environmental change between oxic and anoxic interphase and osmoprotection is probably not surprising given that costal salt marshes are well known for rapid changes in water levels, salinity, temperature, and nutrients.

**Fig. 5:**
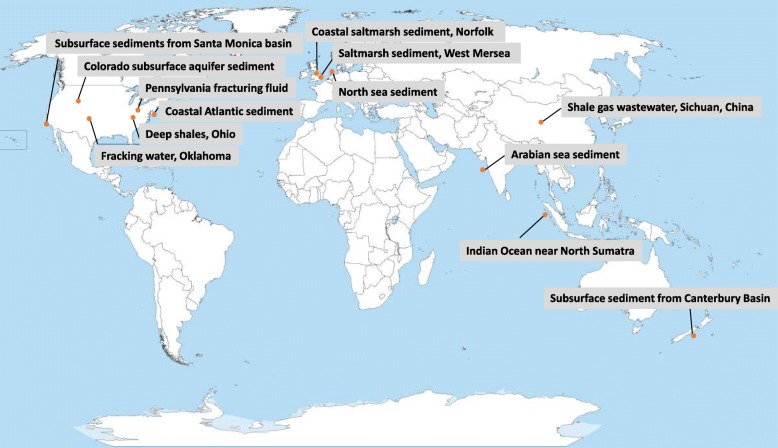
Global distribution of *Canditatus* ‘Betaina sedimentti’ in coastal marine sediments, coastal salt marshes, deep subsurface sediments, and shale gas and oil fracturing waters

To gain a better understanding of the wider distribution of *Candidatus* ‘Betaina sedimentti’, we performed genome mapping by recruiting metagenomic reads using the near-complete genomes assembled from MAGs (bin 4, Table 1). Total number of reads that are mapped to *Candidatus* ‘Betaina sedimentti’ can be highly variable, and not surprisingly, it was detected in high abundance in coastal salt marsh sediments. Reads mapped to this bacterium were also detected in coastal marine sediments in the Indian Ocean, Pacific Ocean, and Atlantic Ocean (Fig. 5). Interestingly, reads mapped to this bacterium are also found in many deep subsurface shale gas and oil fracturing fluids and fracking waters in USA and China, where GBT and its precursor choline are commonly added as chemical additives for hydraulic fracking [24].

Taken together, metabolic reconstruction of this novel bacterial genome obtained from the coastal salt marsh and its global distribution in marine and subsurface sediments reconciles our phylogenetic analyses, supporting the unique features of this sediment-adapted bacterium in the metabolism of the common osmolyte GBT in the *Clostridiales* order.

## Discussion

In this study, through a synthesis of stable isotope probing, high-throughput sequencing, metagenome binning, and metabolic reconstruction, we uncovered a novel family of bacteria involved in methanogenesis from the ubiquitous osmolyte GBT from a coastal salt marsh sediment. GBT is an important osmoprotectant, which is synthesised by many living organisms in response to abiotic stresses such as salt and drought tolerance [25–27]. As a result of its ubiquitous presence in biota, GBT is also commonly found in coastal and marine sediments as well as hypersaline environment and its degradation leads to the release of methane, a potent greenhouse gas [8]. However, the microbes involved in GBT-dependent methanogenesis have not been well studied. Early studies using Bacteria-Archaea co-cultures have demonstrated that methanogenesis from GBT is a two-step process, involving formation of TMA from GBT by the bacterial partner followed by methane production from TMA by the archaeal partner [21, 28]. Such a syntrophic interaction in the GBT-dependent methanogenesis helps to interpret the close association of sulfate reducers and methylotrophic Archaea that is observed in many ecosystems where high osmotic pressure is expected, such as coastal and marine sediments and hydraulic fracturing fluids [29, 30].

The notion that syntrophic interaction between Bacteria and methylotrophic Archaea is a necessity for GBT-dependent methanogenesis has recently been challenged by several independent studies. It becomes clear that some methanogens in the *Methanococcoides* genus can in fact produce methane through direct demethylation of GBT, yielding dimethylglycine as the by-product [9]. Similarly, Ticak et al. [13] has isolated a *Methanolobus* strain from a marsh on the coast of Virginia, USA, which is able to produce methane directly from GBT at a ratio of 1 GBT:0.71 methane. The work presented in this study supported a two-step methanogenesis of GBT through the formation of TMA as a key intermediate (Fig. 1a) in this salt marsh sediment although it is difficult to rule out the possibility of direct demethylation of GBT for methanogenesis. Indeed, TMA formation from other quaternary amine precursors has also been observed in this salt marsh and we have shown previously that TMA can be produced by bacteria from choline fermentation [31].

Early work on salt marsh sediments has shown a strong stimulation of sulfate reduction by the addition of GBT, suggesting that GBT degradation in salt marsh sediments was likely carried out by sulfate reducers [8]. Indeed, we also observed a steady increase in the relative abundance of sulfate reducers in our microcosms amended with GBT (Fig. 1b). In particular, *Desulfobacterium* spp. (family *Desulfobacteraceae*) were enriched in GBT amendment. In the bins that were assigned to *Desulfobacterium*, a complete set of genes required for sulfate reduction is present (Additional file 6: Figure S8). These sulfate reducers do not appear to produce TMA, and the GBT reductase genes are absent in its genome. Interestingly, both King’s study [8] and our microcosm incubation studies showed that the molar conversion of GBT to methane is 1:1 (Fig. 1a), reaching only ~ 44% of the theoretical value. Therefore, it is likely that at least some of the GBT added to the microcosm in these coastal marine sediments was degraded in a TMA-independent pathway. *Desulfobacterium* spp. in this salt marsh appear to oxidise GBT to dimethylglycine using the newly characterised MtgAB methyltransferase [32], similar to other cultivated *Desulfobacterium* strains [12].

Interestingly, our data presented in this study suggest that methanogenesis from GBT in this salt marsh ecosystem relies on the initial degradation of GBT by a novel family of fermentative bacteria as opposed to sulfate reducers. *Candidatus* ‘Betaina sedimentti’ appears prevalent in several ecosystems where high osmotic pressure may be expected, such as the coastal marine sediment, subsurface aquifer sediment, and fracturing fluids from shale gas extraction (Fig. 2). This notion was further supported by mapping published metagenome reads from a range of ecosystems against the genome of *Candidatus* ‘Betaina sedimentti’ (Fig. 5). Reads mapped to *Candidatus* ‘Betaina sedimentti’ were found in coastal sediments in the North Sea sampled after phytoplankton bloom [33] and coastal sediments from the Atlantic, Pacific, and Indian Oceans as well as subsurface fracking fluid in several sites in USA and China [34]. This suggests that GBT may represent an important osmoprotectant as well as a nutrient source for this bacterium to thrive in such ecosystems of high osmosis. Recovery of the near-complete genome of *Candidatus* ‘Betaina sedimentti’ allowed for metabolic reconstruction, which not only confirmed the genetic potential for GBT degradation to TMA via the glycine betaine reductase pathway, but also demonstrated the lack of anaerobic respiration using sulfate or other terminal electron acceptors (Fig. 3). The presence of multiple mechanisms of osmoprotection and an array of two-component systems and oxidative stress responses reconcile our hypothesis that this bacterium may occupy a niche of frequent fluctuation in environmental conditions such as the salt marsh and coastal sediments.

## Conclusions

Combining DNA-stable isotope probing with metagenomics sequencing and assembly allowed the retrieval of near-complete genomes of a novel family of clostridial bacteria involved in GBT degradation in coastal marine sediments. The result presented in this work demonstrated the power of multidisciplinary approaches to uncover metabolic functions in as-yet uncultivated novel environmental microbes.

## Methods

### Environmental sampling and microcosm incubations

Sediment cores were taken from the Stiffkey salt marsh, Norfolk, UK, between October and November 2013. Three sediment cores (10–15 cm in depth) were extracted from the salt marsh, which were transported to the laboratory on the same day and stored overnight at 4 °C before processing in the following morning. A sterilised ruler (70%, v/v ethanol) was used to remove the sediment from the core at five depths (0–0.5, 0.5–1.5, 1.5–4.5, 4.5–7, and 7–10 cm). Prior to DNA stable isotope labelling (SIP) experiments, microcosms were set up in three biological replicates to determine the most active layers for methanogenesis from glycine betaine (GBT) and trimethylamine (TMA). Furthermore, no substrate-added control incubations were set up to determine intrinsic methane formation.

To determine the microbes responsible for methane formation from TMA and GBT by DNA SIP approach, 5 g of sediments from the most active layer (1.5–4.5 cm), mixed with 20 ml sterile sea water (4%, w/v, sea salt from Sigma Aldrich), was incubated in a 125-ml serum vial. Microcosms were set up in three biological replicates by adding ^13^C_3_-TMA, ^12^C_3_-TMA, ^13^C_2_-GBT, or ^12^C-GBT (purchased from Sigma Aldrich), respectively, to a final concentration of 5 mM (Time point 0, T_0_). Microcosms were monitored for methane formation and depletion of substrate at regular intervals until 5 (T1), 50 (T2), and 120–150 (T3) μmol methane per gram of sediment were produced. Three biological replicated microcosms were then destructively sampled and frozen at − 20 °C for subsequent DNA isolation.

### Gas chromatography and ion-exchange chromatography

Quantification of methane in the gas headspace of microcosm vials was achieved using an Agilent 6890 gas chromatograph equipped with a flame ionisation detector [35]. Methane concentrations were calculated based on a calibration curve with methane standards (0.05–2%, v/v). GBT and TMA were quantified by a cation-exchange chromatograph using a Metrosep C4-250 column with a conductivity detector [36]. A 200-μl liquid sample was taken from the microcosm vial, filtered using a 0.22-μm Nylon centrifuge tube filter (Costar, Corning, NY, USA), diluted 1/10 using Milli-Q water, and analysed by ion-exchange chromatography. A standard curve of GBT and TMA was established for each compound, and the data were process using the MagIC Net 3.0 software package (Metrohm).

### DNA isolation, ultracentrifugation, and Miseq sequencing

DNA extractions from unincubated samples (T0) and samples at T1, T2, and T3 were carried out using the FastDNA Spin Kit for Soil (MP Bio Science, Derby, UK). ^13^C-labelled heavy DNA was subsequently separated from unlabelled light ^12^C-DNA using a caesium chloride density gradient ultracentrifugation as described previously [37]. Density formation across 12–14 fractions (400 μl each) was confirmed by measuring refractive indexes using a digital refractometer (Reichert AR2000). DNA was subsequently extracted from caesium chloride using PEG6000 and glycogen as described previously [37].

To determine the microbial populations in ‘heavy’ and ‘light’ fractions, amplicon sequencing was carried out using the primer sets developed by Caporaso et al. [38] which amplifies both bacterial and archaeal 16S rRNA genes (Additional file 4: Table S4). Amplicon sequencing was performed on an Illumina Miseq platform at the University of Warwick Genomics Facility. Amplicon reads were analysed using the QIIME pipeline, and singletons and chimaeras were removed using USEARCH v7 [39] and UCHIME as described previously [31]. OTU binning was carried out against the GreenGenes database.

### Metagenomics and bioinformatics

Metagenomics sequencing was carried out using DNA from ‘heavy’ and ‘light’ fractions of ^13^C_2_-GBT-amended microcosm in three biological replicates at time point 2 (T_2_), together with three replicates of unamended T_0_ samples. Library preparation was performed using the NEBNext® Ultra™ II FS DNA Library Prep Kit for Illumina®, and sequenced on a Hiseq 2500 platform at the Centre for Genomic Research at University of Liverpool, UK, following the manufacturer’s instructions for input DNA < 100 ng. Briefly, to obtain fragments of 100–250 bp, DNA was incubated at 37 °C in the presence of NEBNext Ultra II FS Reaction buffer for 30 min, followed by ligation of fragments to NEBNext Adaptor for Illumina. After clean-up using AmpliClean Magnetic Bead-based PCR Cleanup (Nimagen), fragments got enriched by PCR using NEBNext Multiplex Oligos for Illumina®. Individual libraries were checked for average fragment size distribution and concentration using a high sensitivity DNA assay on a Bioanalyzer 2100 (Agilent), and re-purified using magnetic beads. DNA integrity was re-assessed on the Bioanalyzer 2100 (Agilent), and libraries pooled at equimolar concentration to obtain the desired number of reads per sample. Library size selection (220–600 bp) was carried out using a Pippin Prep (Sage Science) with a 2% (w/v) cassette and the size-selected pooled library sequenced on a Hiseq 2500 platform (Illumina) at the Centre for Genomic Research at the University of Liverpool, UK. Trimming and adaptor removal was performed as follows: Raw Fastq files were trimmed for the presence of Illumina adapter sequences using Cutadapt version 1.2.1 [40]. Option -O 3 was used, to trim the 3' end of any reads which matched the adapter sequence for 3 bp or more. The reads were further trimmed by Sickle version 1.200 applying a minimum window quality score of 20. The numbers of raw reads and trimmed reads for the three replicates were T0 unfractionated DNA (75465480, 67741422, 70020956 vs 75035125, 67383922, 69643146), T2 light fractions (87099132, 45194530, 64994348 vs 86403097, 44959213, 64560423), and T2 heavy fractions (66834850, 82688034, 85068034 vs 66223673, 81966176, 84397710). If reads were shorter than 20 bp after trimming, they were removed.

Quality trimmed metagenomics reads were then assembled using metaSPAdes v3.11.1 [41] and binned with MyCC version MyCC_2017 [42] using default settings. Estimation of genome completeness and contamination was carried out using the CheckM program [43]. Taxonomic assignment of each bin was carried out by submitting bins to the Rapid Annotation using Subsystem Technology (RAST) annotation pipeline (‘Classic RAST’ pipeline). To search for presence of functional genes involved in GBT degradation within the bins, bins were annotated using Prokka (v1.12) and BlastP searches (cutoff 1e−30, > 70% identity, manual check of chromosomal region) were carried out against annotated bins (MAGs) using characterised proteins of GrdH (glycine betaine reductase) of *Peptoclostridium acidaminophilum* (previously known as *Eubacterium acidaminophilum* [44]), MtgB (glycine betaine methyltransferase) of *Desulfitobacterium hafniense* [32], and MttB (trimethylamine methyltransferase) of *Methanosarcina barkeri* [45]. To estimate the distribution of MAGs in public available metagenomes from diverse ecosystems (salt marsh, subsurface shale, marine sediment, etc.), sequence read archive (SRA) runs were downloaded using fastq-dump. The short-read aligner BBMap was used for mapping reads to the *Candidatus* ‘Betaina sedimentti’ genome (bin 4, Table 1) with a minimum identity cutoff of 0.97 (minid = 0.97). Annotation of the genome (bin 4) is shown in Additional file 5: Table S5. An overview of the metagenomes used for recruitment of reads, their IMG/SRA genome identity and accession numbers, and total mapped reads can be found in Additional file 3: Table S3.

Multiple sequence alignment was performed using MUSCLE program in the MEGA7 package, and phylogenetic trees were inferred from sequence alignment using the neighbour-joining statistical method with 500 bootstrap replications [15].

## Additional files

Additional file 1: **Table S1.** The 50 most abundance OTUs in the glycine betaine (GBT)-amended microcosms and their relative abundance in the ‘heavy’, ‘light’ and unfractionated DNA during GBT enrichment. (XLSX 20 kb)

Additional file 2: **Table S2.** Summary of metagenome-assembled genomes (MAGs) from the GBT-stable isotope probing enrichment. (XLSX 20 kb)

Additional file 3: **Table S3.** Metagenomes used for read-recruitment against Candidatus ‘Betaina sedimentti’. (XLSX 13 kb)

Additional file 4: **Table S4.** Samples subjected to amplicon sequencing of 16S rRNA genes in this study. (XLSX 14 kb)

Additional file 5: **Table S5.** Annotation of MAG (Bin 4) by RAST (Rapid Annotation using Subsystem Technology). (XLS 3473 kb)

Additional file 6: **Figure S1.** Microcosm incubation of salt marsh sediments with trimethylamine (TMA). Samples were taken at 234 hr (T1), 307 hr (T2) and 427 hr (T3) for amplicon sequencing of microbial 16S rRNA genes. Methane was quantified using a gas chromatograph and TMA was quantified using an ion exchange chromatograph. Error bars indicate standard deviation from 3-6 biological replicates. **Figure S2.** Phylogenetic analyses of 16S rRNA genes of the sulfate-reducing proteobacterial OTUs that were enriched by GBT. Relative abundance of the *Desulfobacteraceae*, *Desulforomonadaceae* and *Desulfobulbaceae* was plotted during GBT enrichment overtime. GBT addition significantly enriched two OTUs (OTU822440212; OTU167393497) that are affiliated to *Desulfobacterium* of the *Desulfobacteraceae* family. **Figure S3.** Genome characteristics and comparative genomes of two Desulfobacterium MAGs against cultivated representatives. **Figure S4.** Phylogenetic analyses of the archaeal 16S rRNA genes of the *Methanococcoides* OTUs that were enriched by GBT (OTU810315212) and TMA (OTU823373423). Relative abundance of *Methanococcoides* in GBT- and TMA- amended microcosms was plotted during the course of enrichment. **Figure S5.** Genome characteristics and comparative genomes of two *Methanococcoides* MAGs against cultivated representatives. **Figure S6.** Amplicon sequencing analyse of 16S rRNA genes from ‘heavy’ (H) and ‘light’ (L) fractions of DNA-stable isotope probing incubations using a) ^13^C_2_-GBT and b) ^13^C_3_-TMA respectively. All incubations were set up in three biological replicates. **Figure S7.** Phylogenetic analyses of the RpoB showing the evolutionary relationship of *Candidatus* ‘Betaina sedimentti’ in the *Clostridiales* order. Scale bar represents substitution per amino acid position. **Figure S8.** An overview of the metabolic reconstruction of *Desulfobacterium* (Bin 22, Heavy_R3, Table 1). This sulfate-reducing bacterium is enriched by GBT in microcosm incubations (Figure S2). (PDF 2133 kb)

## Data Availability

Read data have been submitted to the Sequence Read Archive (SRA) under the accession numbers SRR7964927, SRR7968363, SRR8068353, and SUB5592648.
