# Supplementary material for: A new family of uncultivated bacteria involved in methanogenesis from the ubiquitous osmolyte glycine betaine in coastal saltmarsh sediments
Source: Microbiome. 2019 Aug 27;7:120. doi: 10.1186/s40168-019-0732-4 (PMC6716910; doi:10.1186/s40168-019-0732-4)
Supplement: Supplementary file 6 — Figure S1. Microcosm incubation of salt marsh sediments with trimethylamine (TMA). Samples were taken at 234 hr (T1), 307 hr (T2) and 427 hr (T3) for amplicon sequencing of microbial 16S rRNA genes. Methane was quantified using a gas chromatograph and TMA was quantified using an ion exchange chromatograph. Error bars indicate standard deviation from 3-6 biological replicates. Figure S2. Phylogenetic analyses of 16S rRNA genes of the sulfate-reducing proteobacterial OTUs that were enriched by GBT. Relative abundance of the Desulfobacteraceae, Desulforomonadaceae and Desulfobulbaceae was plotted during GBT enrichment overtime. GBT addition significantly enriched two OTUs (OTU822440212; OTU167393497) that are affiliated to Desulfobacterium of the Desulfobacteraceae family. Figure S3. Genome characteristics and comparative genomes of two Desulfobacterium MAGs against cultivated representatives. Figure S4. Phylogenetic analyses of the archaeal 16S rRNA genes of the Methanococcoides OTUs that were enriched by GBT (OTU810315212) and TMA (OTU823373423). Relative abundance of Methanococcoides in GBT- and TMA- amended microcosms was plotted during the course of enrichment. Figure S5. Genome characteristics and comparative genomes of two Methanococcoides MAGs against cultivated representatives. Figure S6. Amplicon sequencing analyse of 16S rRNA genes from ‘heavy’ (H) and ‘light’ (L) fractions of DNA-stable isotope probing incubations using a) 13C2-GBT and b) 13C3-TMA respectively. All incubations were set up in three biological replicates. Figure S7. Phylogenetic analyses of the RpoB showing the evolutionary relationship of Candidatus ‘Betaina sedimentti’ in the Clostridiales order. Scale bar represents substitution per amino acid position. Figure S8. An overview of the metabolic reconstruction of Desulfobacterium (Bin 22, Heavy_R3, Table 1). This sulfate-reducing bacterium is enriched by GBT in microcosm incubations (Figure S2). (PDF 2133 kb) [file 40168_2019_732_MOESM6_ESM.pdf]

Figure S1

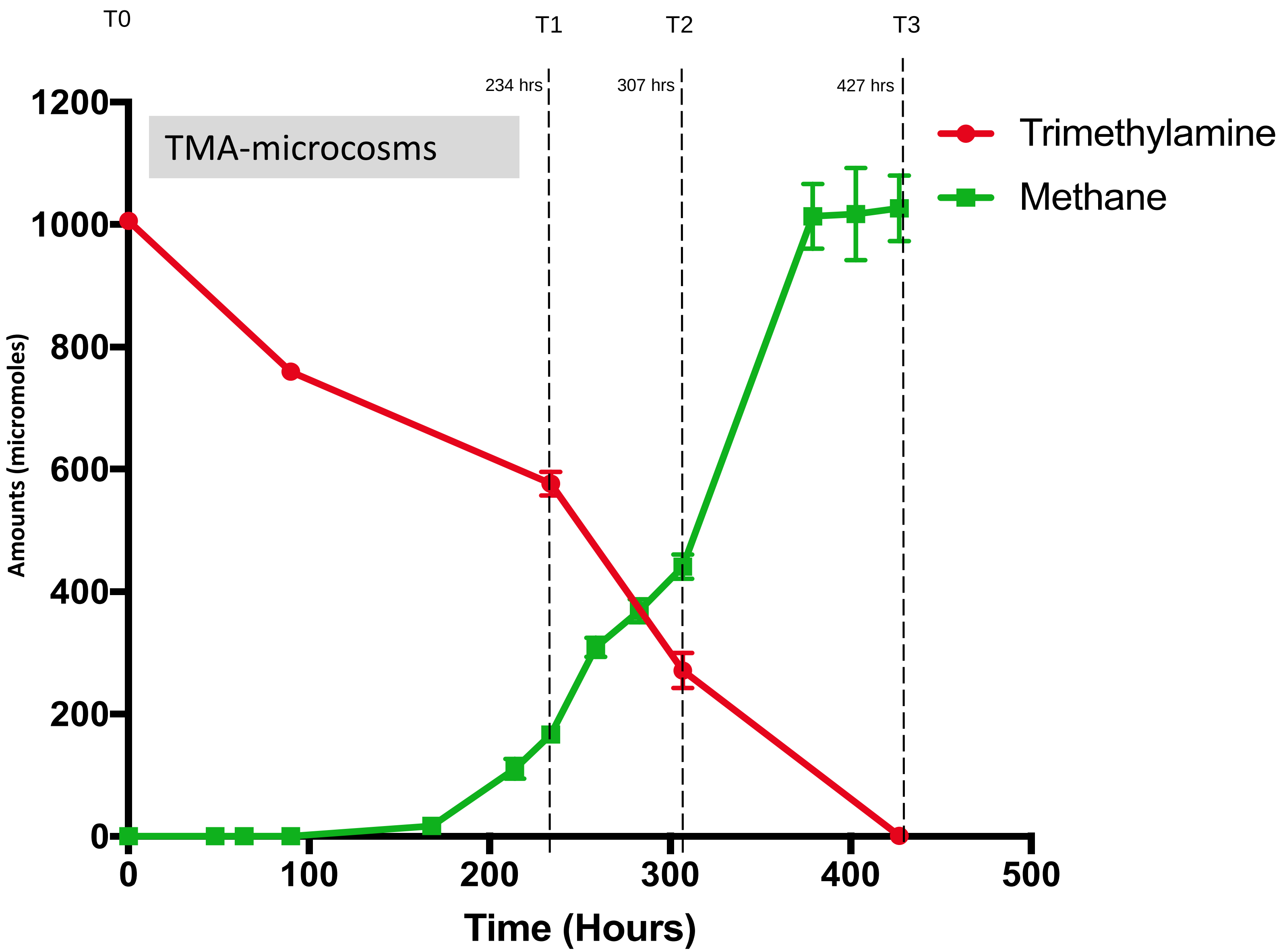
T0
T1
T2
T3
234 hrs
307 hrs
427 hrs
TMA-microcosms
Trimethylamine
Methane
Amounts (micromoles)
Time (Hours)
0
200
400
600
800
1000
1200
100
300
500

Figure S2

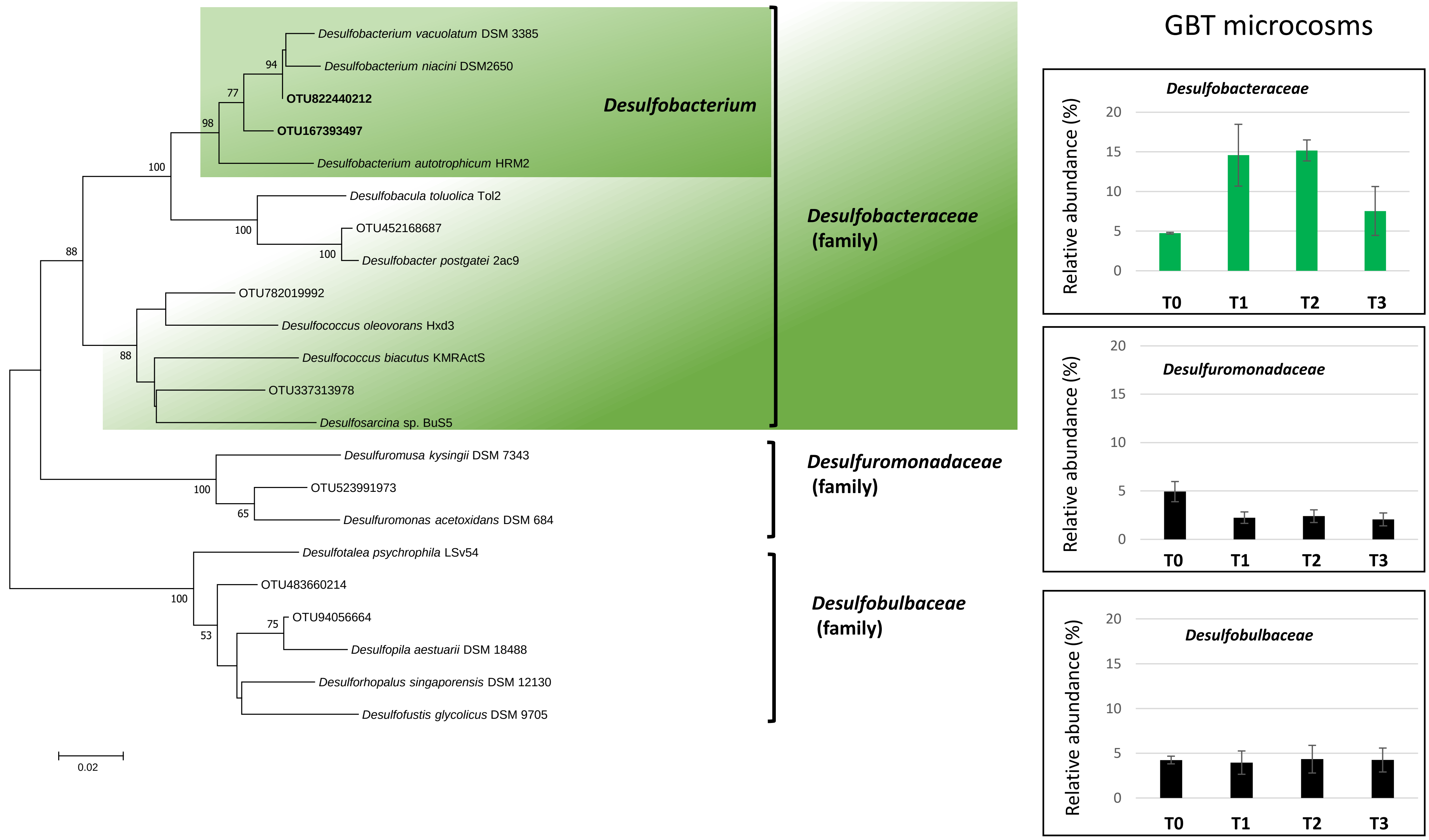

Desulfobacterium vacuolatum DSM 3385
Desulfobacterium niacini DSM2650
OTU822440212
OTU167393497
Desulfobacterium autotrophicum HRM2
Desulfobacterium
Desulfobacula toluolica Tol2
OTU452168687
Desulfobacter postgatei 2ac9
OTU782019992
Desulfococcus oleovorans Hxd3
Desulfococcus biacutus KMRActS
OTU337313978
Desulfosarcina sp. BuS5
Desulfobacteraceae (family)
Desulfuromusa kysingii DSM 7343
OTU523991973
Desulfuromonas acetoxidans DSM 684
Desulfuromonadaceae (family)
Desulfotalea psychrophila LSv54
OTU483660214
OTU94056664
Desulfopila aestuarii DSM 18488
Desulforhopalus singaporensis DSM 12130
Desulfofustis glycolicus DSM 9705
Desulfobulbaceae (family)
94
77
98
100
100
100
88
88
100
65
100
53
75
0.02
GBT microcosms
Desulfobacteraceae
Desulfuromonadaceae
Desulfobulbaceae
Relative abundance (%)
0
5
10
15
20
T0
T1
T2
T3

Genome characteristics of two *Desulfobacterium*-related population genomes extracted from metagenomes and two *Desulfobacterium* genomes

Figure S3

Organism	IMG genome ID	Genome size (Number of total bases)	Gene count (Number of total genes)	CDS count (Number of CDS genes)	rRNA count (Number of rRNA genes)	Protein coding genes with function prediction	Protein coding genes without function prediction
13C Heavy 2 Bin 21	-	6,489,886			26	3443	2156
13C Heavy 3 Bin 22	-	6,624,919			34	3680	2217
D. autotrophicum DMS 3383	643692021	5,657,782	5012	4943	69	3552	1391
D. vacuolatum DSM 3385	2681813565	5,035,799	4183	4101	82	3302	799

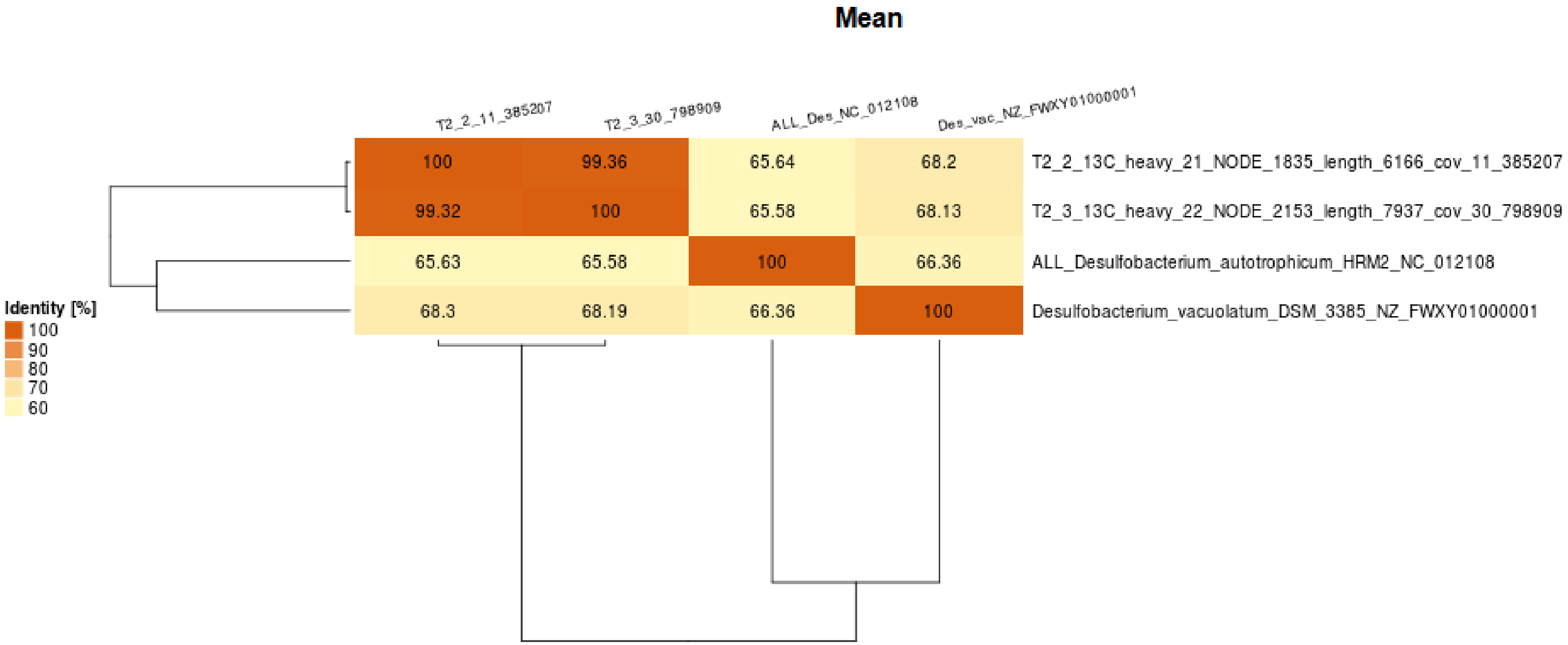

Figure S4

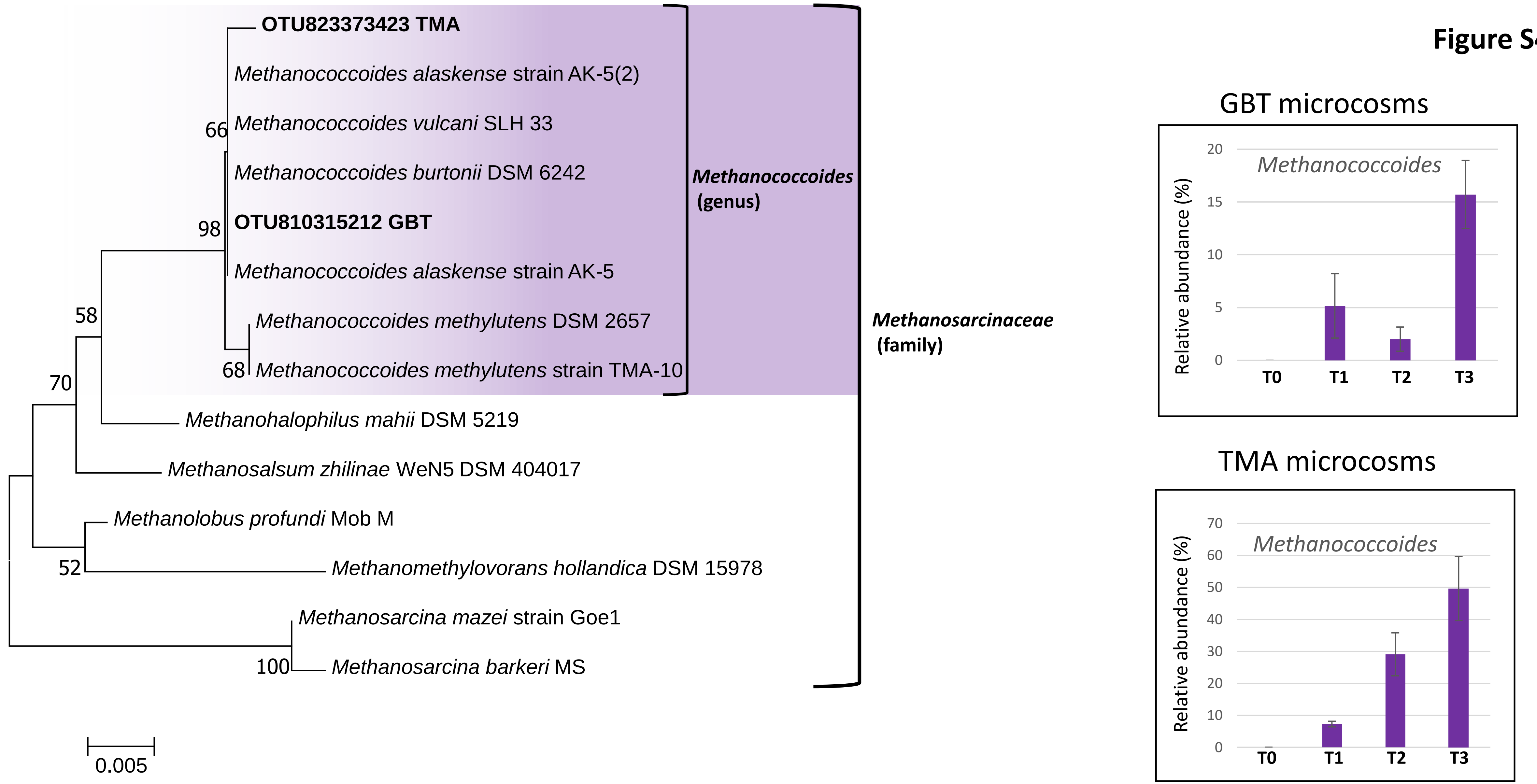

OTU823373423 TMA
Methanococcoides alaskense strain AK-5(2)
Methanococcoides vulcani SLH 33
Methanococcoides burtonii DSM 6242
OTU810315212 GBT
Methanococcoides alaskense strain AK-5
Methanococcoides methylutens DSM 2657
Methanococcoides methylutens strain TMA-10
Methanohalophilus mahii DSM 5219
Methanosalsum zhilinae WeN5 DSM 404017
Methanolobus profundi Mob M
Methanomethylovorans hollandica DSM 15978
Methanosarcina mazei strain Goe1
Methanosarcina barkeri MS
66
98
58
70
68
52
100
0.005
Methanococcoides (genus)
Methanosarcinaceae (family)
GBT microcosms
Methanococcoides
Relative abundance (%)
0
5
10
15
20
T0
T1
T2
T3
TMA microcosms
Methanococcoides
Relative abundance (%)
0
10
20
30
40
50
60
70
T0
T1
T2
T3

Figure S5

Genome characteristics of two *Methanococcoides*-related population genomes extracted from metagenomes and three *Methanococcoides* genomes

Organism	IMG genome ID	Genome size (Number of total bases)	Gene count (Number of total genes)	CDS count (Number of CDS genes)	rRNA count (Number of rRNA genes)	Protein coding genes with function prediction	Protein coding genes without function prediction
13C Heavy 2 Bin 7	-	1,564,734			24	1,105	378
13C Light 2 Bin 10	-	924,130			14	725	186
M. vulcani SLH 33	2642422537	2,314,846	2,357	2,290	67	1,712	578
M. burtonii DSM 6242	637000161	2,575,032	2,506	2,431	75	1,566	865
M. methylutens DSM 2657	2630968972	2,508,511	2,527	2,462	10	1,805	657

Average amino acid identity

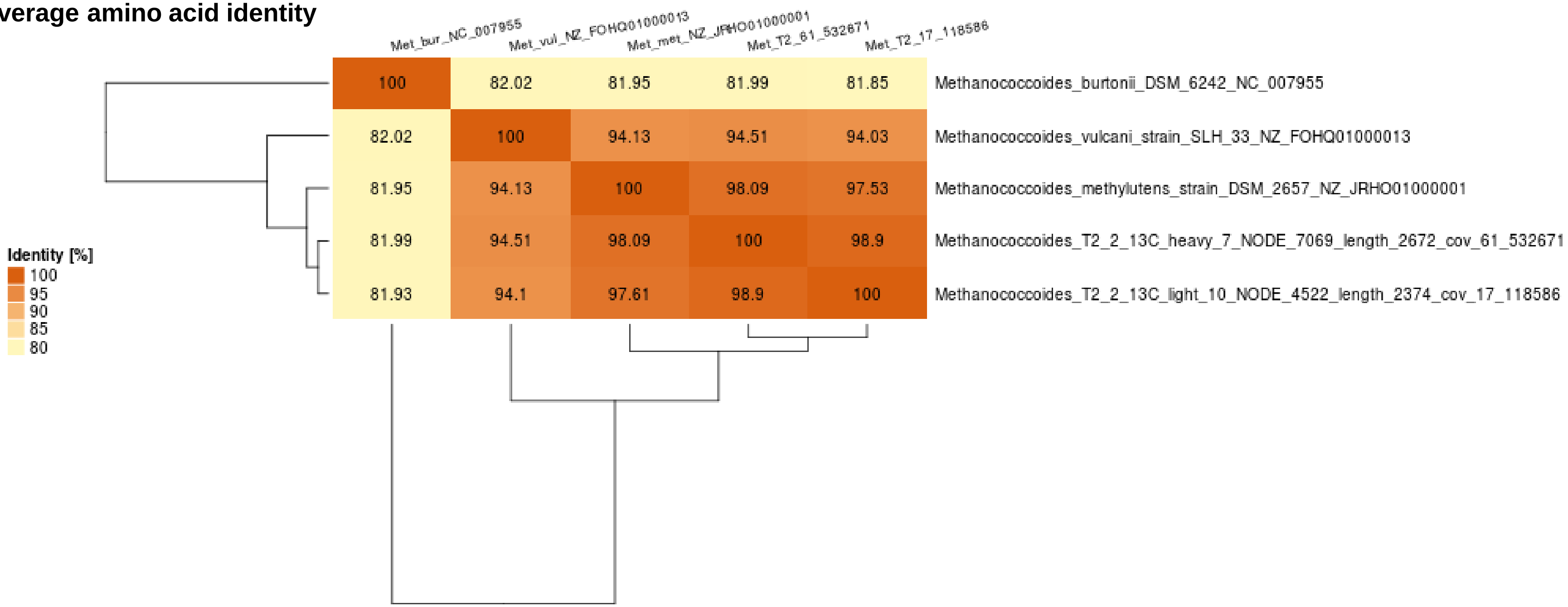

Figure S6

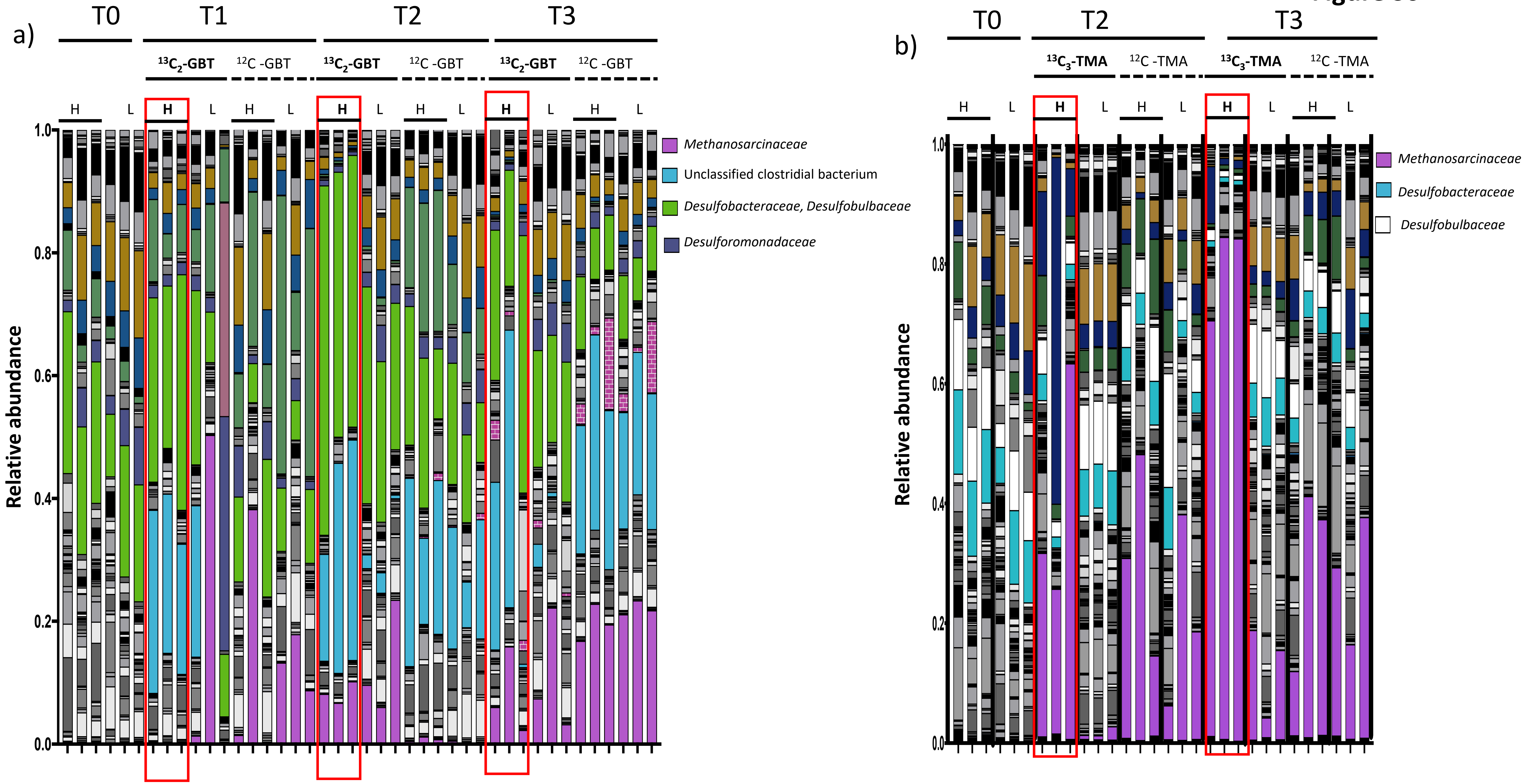
a)
T0
T1
T2
T3
13C2-GBT
12C -GBT
13C2-GBT
12C -GBT
13C2-GBT
12C -GBT
H L H L H L H L H L H L H L
Relative abundance
1.0
0.8
0.6
0.4
0.2
0.0
Methanosarcinaceae
Unclassified clostridial bacterium
Desulfobacteraceae, Desulfobulbaceae
Desulforomonadaceae
b)
T0
T2
T3
13C3-TMA
12C -TMA
13C3-TMA
12C -TMA
H L H L H L H L H L
Relative abundance
1.0
0.8
0.6
0.4
0.2
0.0
Methanosarcinaceae
Desulfobacteraceae
Desulfobulbaceae

Figure S7

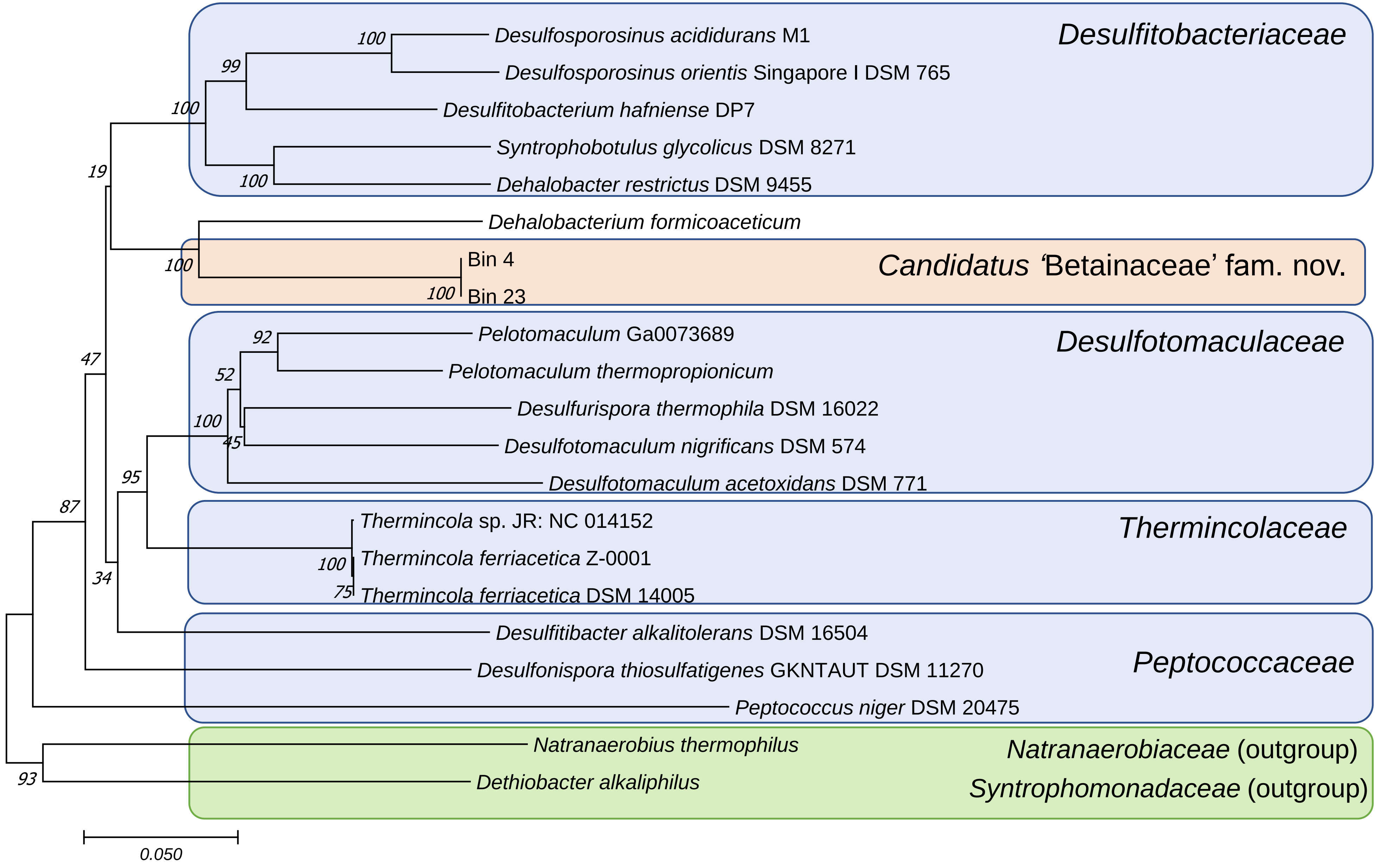
Desulfitobacteriaceae
Desulfosporosinus acididurans M1
Desulfosporosinus orientis Singapore I DSM 765
Desulfitobacterium hafniense DP7
Syntrophobotulus glycolicus DSM 8271
Dehalobacter restrictus DSM 9455
Dehalobacterium formicoaceticum
Candidatus 'Betainaceae' fam. nov.
Bin 4
Bin 23
Desulfotomaculaceae
Pelotomaculum Ga0073689
Pelotomaculum thermopropionicum
Desulfurispora thermophila DSM 16022
Desulfotomaculum nigrificans DSM 574
Desulfotomaculum acetoxidans DSM 771
Thermincolaceae
Thermincola sp. JR: NC 014152
Thermincola ferriacetica Z-0001
Thermincola ferriacetica DSM 14005
Peptococcaceae
Desulfitibacter alkalitolerans DSM 16504
Desulfonispora thiosulfatigenes GKNTAUT DSM 11270
Peptococcus niger DSM 20475
Natranaerobiaceae (outgroup)
Syntrophomonadaceae (outgroup)
Natranaerobius thermophilus
Dethiobacter alkaliphilus
100
99
100
19
100
100
100
92
52
100
45
47
95
87
100
75
34
93
0.050

Figure S8

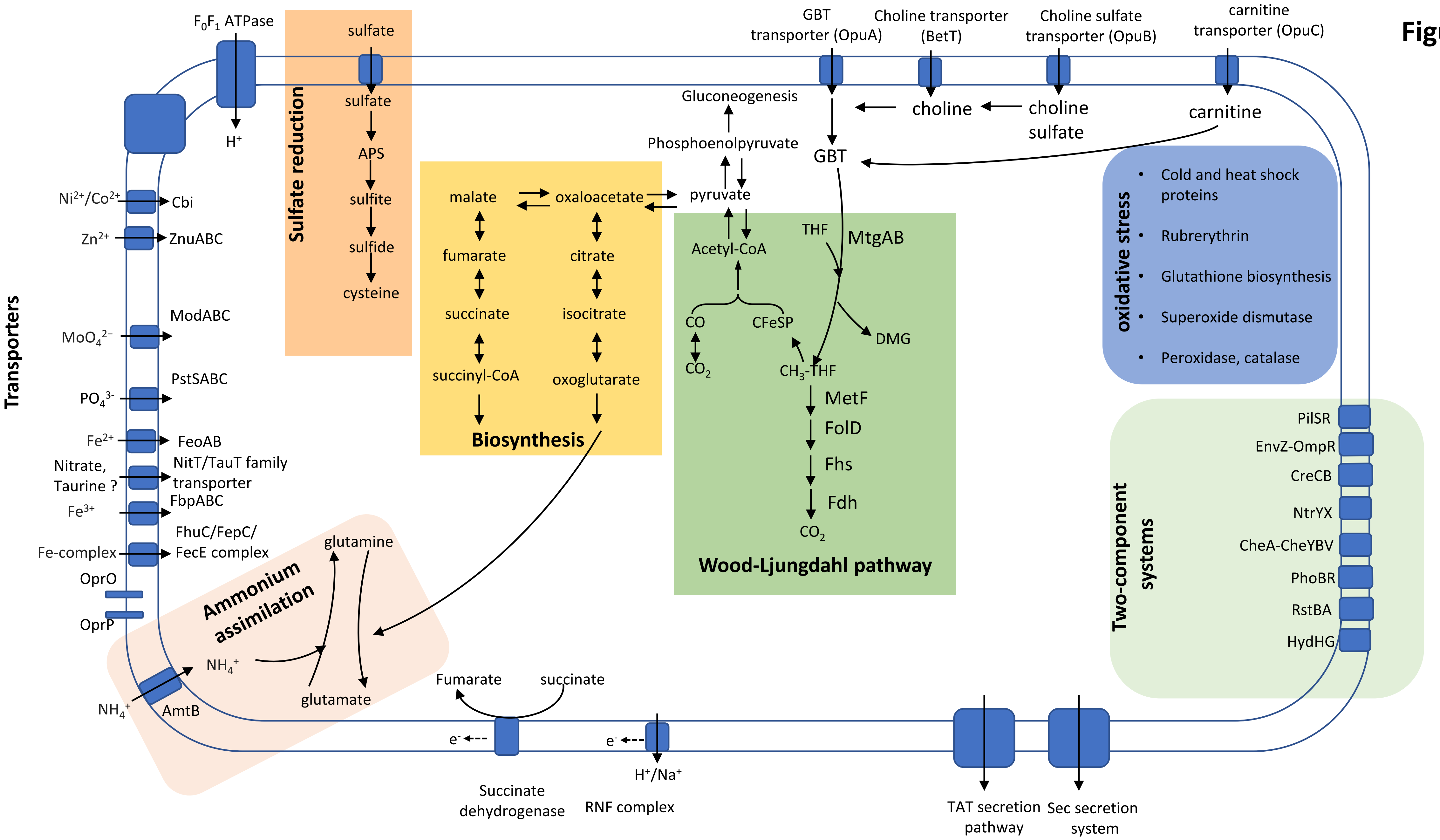

F0F1 ATPase
H+
sulfate
Sulfate reduction
sulfate
APS
sulfite
sulfide
cysteine
GBT transporter (OpuA)
Choline transporter (BetT)
Choline sulfate transporter (OpuB)
carnitine transporter (OpuC)
Gluconeogenesis
Phosphoenolpyruvate
choline
choline sulfate
carnitine
GBT
malate
oxaloacetate
pyruvate
fumarate
citrate
succinate
isocitrate
succinyl-CoA
oxoglutarate
Biosynthesis
Acetyl-CoA
THF
MtgAB
CO
CFeSP
CO2
CH3-THF
DMG
MetF
FolD
Fhs
Fdh
CO2
Wood-Ljungdahl pathway
oxidative stress
Cold and heat shock proteins
Rubrerythrin
Glutathione biosynthesis
Superoxide dismutase
Peroxidase, catalase
Two-component systems
PilSR
EnvZ-OmpR
CreCB
NtrYX
CheA-CheYBV
PhoBR
RstBA
HydHG
Transporters
Ni2+/Co2+
Cbi
Zn2+
ZnuABC
ModABC
MoO42−
PstSABC
PO43-
Fe2+
FeoAB
Nitrate, Taurine ?
NitT/TauT family transporter
FbpABC
Fe3+
FhuC/FepC/ FecE complex
Fe-complex
OprO
OprP
Ammonium assimilation
glutamine
NH4+
glutamate
NH4+
AmtB
Fumarate
succinate
e-
e-
H+/Na+
Succinate dehydrogenase
RNF complex
TAT secretion pathway
Sec secretion system
